# Emergency medical services in the Limpopo province: Is there a need for change and is trauma different?

**DOI:** 10.4102/safp.v68i1.6227

**Published:** 2026-01-14

**Authors:** Samson N. Phaleng, Timothy C. Hardcastle

**Affiliations:** 1 Discipline of General Surgery, School of Medicine, College of Health Sciences, University of KwaZulu-Natal, Durban, South Africa

**Keywords:** EMS, trauma, trauma systems, response time, patient treatment time, mission time

## Abstract

**Background:**

Prehospital emergency medical services (EMS) may reduce morbidity and mortality. Trauma systems may improve survival. The objectives of this study were to determine response times of primary and secondary calls in the Limpopo province and to compare primary and secondary calls with international and South African standards. The goal is to make recommendations on improving policies governing the transportation of emergency patients, specifically addressing the transfer of major and polytrauma patients to tertiary facilities.

**Methods:**

A retrospective observational cross-sectional study of EMS transports to and from three EMS stations in the Limpopo province between 01 January 2020 and 31 December 2020. Data collection included demographics, medical category of patients, response, patient treatment and mission times.

**Results:**

The study included 1040 patients, with a similar distribution of male to female, most (62%) aged between 16 and 54 years. Medical cases constituted 30.5%, followed by maternity (25.6%), then trauma (20.7%). Primary calls constituted 79.9%, while secondary calls encompassed 20.1%. The mean response time of all patients was 28 min. The mean response time for trauma patients was 40 min, while for non-trauma patients, it was 24 min. In linear regression analysis, the type of call was found to be statistically significant in predicting response time with a *p*-value of 0.022.

**Conclusion:**

The mean response time overall and for trauma is far longer than international standards and longer than for non-trauma patients. Efforts should aim to reduce response time for trauma patients.

**Contribution:**

The research highlights the difference in response between trauma and non-trauma cases in a rural setting.

## Introduction

Trauma systems improve the survival rates of trauma patients. Trauma registries represent an integral and essential part of the system to document the epidemiology, management and outcome of trauma patients.^1,2^ Emergency medical services (EMS) provide a spectrum of urgent medical care that includes initial prehospital critical interventions and transportation, ending with handover to continued care in emergency departments, especially in low- and middle-income countries (LMICs).^3^ The systems have been shown to reduce mortality.^4^ Timely prehospital care is key to improving outcomes in time-sensitive injuries in sub-Saharan Africa (SSA).^5^ In general, trauma patients in LMICs requiring interhospital transfer took longer to reach the referral hospital than direct transfers (0.56–37.91 h).^6^

Many LMICs lack components of formal trauma systems, including prehospital EMS, provider education and patient triage, transfer and management protocols.^4^ In Tanzania, major barriers identified in the implementation of community-based or hospital EMS included the financial status of patients and limitations in healthcare insurance and the level of technical credibility of layperson first responders.^7^ In Uganda, there is an unstructured EMS impaired by a lack of national policy, guidelines and standards, funding for medical products and coordination. The police, as well as private for-profit and non-profit agents, provided prehospital care services.^8^

South Africa (SA) currently falls within the World Health Organization trauma system classification of maturity at level 2 (prehospital resources available, identifiable health personnel and with roles of various health facilities clear, but no formal trauma system); however, different situations prevail across the various provinces. There are seven specialised trauma centres spread across the country, with immediately available surgical facilities, and yet there is no formal national trauma system.^4,9^ In Cape Town, a barrier to EMS access included a lack of telephone access and no universal emergency number, which is now corrected. Also, equipment was thought to be a barrier, including ventilators, IV pumps, pulse oximetry and cardiac monitors.^10^ A study in the KwaZulu-Natal (KZN) province by Govender found that the response to obstetric patients was delayed by a lack of ambulances and obstetric equipment.^11^ Also in KZN, Aschokcoomar, et al. found problems such as lack of essential equipment, dysfunctional equipment and unsterile procedures and concluded that EMS providers in the province lacked preparedness to manage interfacility neonatal transfers.^12^ The Limpopo province is an under-researched and under-resourced province where no previous EMS research studies were conducted. The objectives of this study were: (1) to determine response times of primary and secondary calls in the Limpopo province, (2) to compare primary and secondary calls with international and SA standards and then (3) to make recommendations on improving policies governing transportation of emergency patients, specifically addressing the transfer of major and polytrauma patients to tertiary facilities.^13^

## Research methods and design

This was a retrospective cross-sectional study of all patients, including trauma, from 01 January 2020 to 31 December 2020.

### Setting

This study was conducted at the three largest EMS stations in the Limpopo province, namely Mankweng, Mokopane and Philadelphia, in the three largest of the five districts, and this was out of 57 EMS stations in the province, most of which are small stations.

### Participants

Patients of all ages of different categories who were transported by EMS during the above-mentioned study period, which was the time period approved by the Ethics and Provincial Research Committee as part of a PhD study process. The focus was on trauma cases for the detailed analysis.

### Variables

Data were obtained from patient record forms (PRF) and accessed manually, collating demographics, clinical categories, response, treatment and mission times. Response time is from the time the call was received to arrival at the scene. Treatment time is the time at the scene to arrival at the treatment facility. Mission time is the time the call was received to the time the ambulance is available for the next call.

### Statistical analysis

Descriptive statistics were calculated to summarise the demographic and clinical characteristics of the study participants. Frequencies and percentages were generated for the categorical variables (e.g. sex, age group, type of call, medical category, mechanism of trauma and diagnosis). Descriptive measures of response time, patient treatment time and total mission time were calculated including mean, median, standard deviation (s.d.), minimum, maximum and range. Moreover, a boxplot of the response time was developed to determine the operational distribution and assess for outliers. The box plot enabled the observation of cases where response times were longer with atypical delays and variability with respect to operational response. Furthermore, a linear regression analysis was conducted to evaluate the factors that significantly predicted response times. The response time was the dependent variable, while age, sex, type of call, mechanism of trauma, medical category and diagnosis were the independent variables. An independent samples *t*-test was carried out to analyse response times for primary and secondary calls and for trauma and non-trauma cases. This inferential test examined if the type of call had a statistically significant effect on the response time for EMS. All data were analysed using SPSS version 30, and the statistical significance was set at *p* < 0.05. The trauma subset was further compared with the other non-trauma case types, for example, medical and maternity, in more detail.

### Ethical considerations

Ethics approval was obtained from the Biomedical Research Ethics Committee (BREC) (00003999/2022) of the University of KwaZulu-Natal (UKZN) and the Pietersburg Mankweng Research Ethics Committee from the Limpopo province (No. 00003999/2022 and REC 300408006).

## Results

A total of 1040 patients were analysed with a similar distribution for male (49.8%) and female (50.1%) patients. Most of the patients (62.9%) were between 16 and 54 years. This is detailed in Table 1. While trauma was only 20.7% of the workload, it was dominated by head and orthopaedic injuries, in keeping with a blunt trauma dominance.

**TABLE 1:** Demographic and clinical characteristics of patients transported with the emergency medical services system (*N* = 1040).

Variable	*n*	%
**Sex**		
Male	518	49.80
Female	522	50.10
**Age (years)**		
≤ 15	176	16.90
16–54	655	62.90
≥ 55	210	20.20
**Type of call**		
Primary	832	79.90
Secondary	209	20.10
**Medical category**		
Medical	317	30.50
Maternity	271	26.00
Trauma	215	20.70
Surgical	112	10.80
Paediatrics	93	8.90
Gynaecology	21	2.00
Psychiatry	12	1.20
**Mechanism of trauma**		
Assault	101	49.20
Motor vehicle crash	46	22.43
Pedestrian-vehicle crash	19	9.30
Fall	39	19.03
**Diagnosis**		
Medical	322	31.00
Maternity	266	25.60
Surgical	111	10.70
Paediatrics	96	9.20
General body injuries	75	7.20
Head injury	67	6.40
Orthopaedic injuries	43	4.10
Gynaecology	20	1.90
Stab abdomen	12	1.20
Psychiatry	12	1.20
Chest injury	11	1.10
Multiple stabs	2.0	1.10
Stab neck	2.0	1.10
Gunshot abdomen	1	0.10

Primary calls constituted 79.9%, while secondary calls encompassed 20.1%. With primary calls, 83.0% were non-trauma, while with secondary calls, 69.4% were non-trauma. Most of the patients fell into the medical category (30.5%), followed by maternity (25.6%) and then the trauma (20.7%) group. Trauma cases were mostly caused by assault (49.2%), followed by motor vehicle crash (22.4%). Further assessment of the trauma cohort was undertaken.

The average response time for all patients was 28 min. The average patient treatment time (rounded to the nearest minute) was 54 min, while the mission time was 2 h 37 min. This is illustrated in Table 2 with the detailed times listed. The mean response time for primary calls was 25 min, while the mean response time for secondary calls was 37 min. Student’s *t*-test produced a *t*-value of −1.740 with 1035 degrees of freedom and a *p*-value of < 0.001, indicating a statistically significant difference in response times for the two call types. The mean mission time for secondary calls was 7 h. This is shown in Table 2 and Table 4. Table 3 reveals that the response time for trauma patients was longer at 39 min when compared with non-trauma patients (24 min), while the mission time for trauma patients was 3 h 07 min as compared with 2 h 30 min for non-trauma patients. In Table 5, the Student’s *t*-test shows a statistically significant difference in response times between trauma and non-trauma cases, and trauma cases had a mean delay of 15 min.

**TABLE 2:** Descriptive statistics of response, patient treatment and mission times of patients transported with the emergency medical service system.

Descriptive statistics	Response time	Patient treatment time	Mission time
Mean	28 min	54 min	2 h 37 min
Median	20 min	36 min	1 h 20 min
Standard deviation	1 h 30 min	56 min	2 h 48 min
Minimum	01 min	03 min	02 min
Maximum	35 h	12 h 10 min	19 h
Range	34 h 59 min	12 h 07 min	18 h 58 min

min, minutes; h, hour.

**TABLE 3:** Descriptive statistics of response times, patient treatment time and mission times of trauma and non-trauma patients with the emergency medical service system.

Variable	Response time (mean)	Patient treatment time (mean)	Mission time (mean)
Trauma	39 min	64 min	3 h 07 min
Non-trauma	24 min	51 min	2 h 30 min

min, minutes; h, hour.

**TABLE 4:** Independent samples *t*-test comparing the response time based on the type of call.

Test variable	*T*	*df*	95% CI		*p*-value
			Lower	Power	
Response time	- 1.740	1035 -	- 1592 742	- 318 861	< 0.001 -

*df*, degrees of freedom; CI, confidence interval; *T*, *T*-statistic.

In Table 5, the Student’s *t*-test shows a statistically significant difference in response times between trauma and non-trauma cases, and trauma cases had a mean delay of 15 min.

**TABLE 5:** Independent samples *t*-test comparing the response time between trauma and non-trauma cases.

Test variable	*T*	*df*	*p*-value	Mean difference (min)
Response time	2.240	1035	0.025	15

*T, T*-statistic; *df*, degrees of freedom.

Using linear regression analysis, as illustrated in Table 6, only the type of call (whether primary or secondary) was known to be statistically significant in predicting response time (*p*-value of 0.022).

**TABLE 6:** Linear regression showing the factors associated with the response time of the emergency medical service system.

Predictors	*β*	*p*-value
Sex	−0.019	0.570
Age	−0.028	0.396
Type of call	0.073	0.022
Mechanism of trauma	0.056	0.181
Medical category	0.194	0.329
Diagnosis	0.255	0.191

*β*, Beta coefficient.

## Discussion

Providing timely access to EMS in SA remains elusive in both urban and rural environments.^14^ Emergency medical service in most provinces is still developing and not without challenges.^8^ The SA healthcare system is built on three tiers (primary, secondary and tertiary or quaternary) characterised by multiple health facilities sited more for political and historical reasons rather than modern population density and demand.^15^ The majority of patients transported with EMS were medical patients, followed by maternity and then trauma. This concurs with the general South African quadruple burden of disease, the human immunodeficiency virus (HIV) or acquired immunodeficiency syndrome (AIDS) epidemic, with a high burden of tuberculosis (TB), high maternal mortality and high levels of trauma and violence, in addition to non-communicable diseases.^16^

### The response time interval

The mean response time in the study was 28 min. This is 3.5 times the international 8 min-norm in a metropolitan area and almost twice the SA standards for response to emergency cases of 15 min.^17^ The mean response time for trauma patients was 39 min as compared with 24 min for non-trauma patients. The mean response time for secondary calls was even longer, at 37 min, than for primary calls.

### The patient treatment time

The mean patient treatment time was 54 min. This is also higher than SA standards of 40 min. The treatment time was 64 min for trauma patients as compared with 51 min for non-trauma patients.

### The mission time

The mean mission time in the study was 2 h 37 min. This is slightly higher than 2 h expected in a metropolitan setting. The mean mission time was 3 h 7 min for trauma patients as compared with 2 h 29 min for non-trauma patients. The mean mission time for secondary calls was 7 h.

The response and mission times were higher than both SA and international standards and more so with secondary calls. The process of transporting patients can be described as challenging and hampered by many delays. These can include delays in contacting EMS control centres (communication issues), a shortage of ambulances and, with inter-facility transfers, the need to confirm a receiving doctor before transporting the patient, among other issues. This situation is applicable to most under-resourced and underprivileged areas such as the Limpopo province.^18^ In general, interfacility transfers have longer delays due to a shortage of ambulances, human resource issues (as they generally require higher care levels and there are limited numbers of advanced care practitioners) and the prioritisation of primary calls.^19^

The mean response time of 37 min for secondary calls was longer than the EMS trauma response time reported by Mahama et al. (17 min average), with recorded times of 10 min for urban cases and longer than the 12 min in suburban areas reported by Karagholi et al.^20,21^ The mean response time for trauma versus non-trauma patients (39 min vs 24 min) was consistent with findings by Avidar et al., who also showed longer trauma case times (6 min vs 3.5 min).^22^ The response times, patient treatment times and mission times were longer for trauma patients because of the complexities of the injuries and stabilisation strategies that are often employed.

### Limitations

This study, by being retrospective, is subject to selection bias, and there was no electronic record keeping requiring manual data collection, which may have led to missed case reports. The empirical choice of three ambulance bases and not all the ambulance bases in the province may also have introduced further selection bias.

## Conclusion

The response times and mission times are generally longer than recommended standards. The mean response time for primary calls was 25 min, while the mean response time for secondary calls was 37 min. Compared with international standards of 8 min and acceptable SA standards of 12 min, they are higher. The mean response time for severe trauma patients was 39 min, which is far longer than any acceptable standards. The SA EMS system is now experiencing multiple challenges that can be addressed by increased staff numbers and vehicles with optimal centralised control with effective communication systems and call prioritisation, which have been shown to reduce mortality. The EMS system in Limpopo is currently not adequately fulfilling its role, which is to ensure timely response and transfer to hospital of emergency cases, and this requires urgent intervention by the National Department of Health. Future research should include how implementation barriers of EMS services impact the outcome of trauma patients.
